# SOFCs integrated with SMES under dynamic power control using Chernobyl disaster optimizer

**DOI:** 10.1038/s41598-025-86493-y

**Published:** 2025-01-24

**Authors:** Sameh I. Selem, Attia A. El-Fergany, Eid A. Gouda, Mohamed F. Kotb, Islam Ismael

**Affiliations:** 1 Department of Electric Power and Machines, Faculty of Engineering, Zagazig University, Zagazig, 44519 Egypt; 2 Department of Electrical Engineering, Faculty of Engineering, Mansoura University, Mansoura, Egypt; 3 Faculty of Engineering, Mansoura National University, Mansoura, Egypt

**Keywords:** Solid oxide fuel cells, Unknown parameters, Steady-state model, Dynamic performance, Optimization methods, Energy storage device, Engineering, Electrical and electronic engineering, Energy infrastructure

## Abstract

The current study uses the Chernobyl disaster optimizer (CDO), a new metaheuristic optimizer, to identify the seven unknown parameters of solid oxide fuel cells (SOFCs). The procedures of the CDO is based on physical behavior of the elaborated radiations from the well-known Chernobyl disaster according to their mass, speed, frequency, and degree of ionization. The sum of square errors (SMSE) among the estimated and the real measured output voltage datasets of SOFCs is minimized employing the CDO. Set of boundaries of the SOFC’s process is taken into consideration with the problem formulation. SOFCs stack’s model is examined at 800^ο^C and 900^ο^C and its performance is confirmed. The CDO extracts more precise SOFCs’ parameters compared to other competitors. The CDO’s convergence patterns and the SOFCs unit’s performance are studied and proved at steady-state by comparing its results to a number of recognized algorithms under varied operating scenarios. A significant SMSE’s values of 3.46 µV^2^ and 7.38 µV^2^ are attained at 800^ο^C and 900^ο^C, respectively by the CDO. As a result, the polarization principal curves of the measured and estimated voltage datasets are checked and verified with very close matching. The dynamic behavior of the SOFCs stack is examined in relation to direct load, electric networks, and superconducting magnetic energy storage devices (SMES) for additional validation and illustration. The role of the SOFCs stack in controlling the active and reactive power delivered to the network and direct load is investigated using two controllers: one to control the inverter, which converts the SOFC’s dc output to the main network, and the other to control the SMES. The Simulink/MATLAB environment is used to indicate the validity of the proposed framework under both steady-state and dynamical conditions. The comprehensive assessments show that the CDO capabilities are very effective when used with microgrids.

## Introduction

The precision of defining the solid oxide fuel cells (SOFCs)’ parameters plays an important role in providing a consistent plan when integrated with the energy storage system^1^. Also, it highly affects the thermal gradient within the cells under the steady-state and dynamic performances. An intense competition among several optimizers is required to acquire the most accurate SOFCs’ parameters possible^2,3^.

### Motivation of the current effort

The ongoing decline of traditional fossil fuels, their inefficiency, and their negative effects on the environment led to the search for more environmentally friendly, more efficient, and less polluting alternatives^4–6^. Fuel cells (FCs), photovoltaic units, wind energy, microturbines, etc. have been developed as renewable distributed energy resources (DER) and represent nearly 26% of the total generated energy^3,7^. DER can be integrated to the main network not only to compensate the shortcomings of the conventional resources but also to increase power system reliability and to make distribution system active^6^. Hydrogen has a main role in obtaining net-zero emissions all over the world and reducing 80 Giga tons of $\:{CO}_{2}$by 2050^8^. Selecting the best DER mainly depends on the environment, topographic anatomy, and fuel availability. FCs have been employed for electric vehicles due to its simplicity, integrated structure, and great efficiency^9,10^. FCs can be considered as one of the best alternatives of conventional generating units/technologies. Its electric efficiency is greater than three times internal combustion engines^11^. The multiple benefits of SOFCs units, including low emissions, efficiency, and fuel lightweight, inspire researchers to raise its performance and reduce its cost so that it can be widely used in industry^3,12,13^. Likewise, SOFCs match many types of fuels starting from hydrogen to carbon oxide and hydrocarbons which is able to produce large amounts of heat that can be utilized while it is operated without vibration^2,14^. The compulsory high operating temperature used for SOFCs stack requires special materials with high cost which is considered the main drawback of the SOFC. The modelling of SOFCs stack is a complicated issue because of its nonlinear and non-convex nature^2,15–17^. An accurate modelling for SOFCs became a challenge to know its actual behavior and performance at different operating conditions^2,3,18^. These aforementioned challenges lead authors to address this subject.

### Literature review

FCs convert fuel chemical energy to electrical forms that look like batteries but cannot be stored. If rich hydrogen fuel is exposed to a catalytic anode, oxidant (often air), and electrolyte, it will undergo oxidation and reduction processes, generate potential voltage, and release some water and heat^19,20^. Solar systems are capable of producing hydrogen with 37% efficiency, making it one of the most efficient energy sources^4^. FCs have higher efficiency, lower fuel oxidation temperature, lower $\:{CO}_{2}$emissions, no noise and vibration, robust and low maintenance than conventional resources. FCs efficiencies range between 36% up to 60% based on its kind and arrangement and can be increased to 85% by using different heat retrieval methods^6^. FCs can use different types of fuel which guarantee more availability. FCs can be classified based on the used electrolyte and the operating temperatures. The main four types are proton exchange membrane FCs (PEMFCs), phosphoric acid FCs (PAFCs), molten carbonate FCs (MCFCs) and SOFCs that can be operated at around $\:{80}^{\text{o}}\text{C}$, $\:{200}^{\text{o}}\text{C}$, $\:{650}^{\text{o}}\text{C}$ and $\:{900}^{\text{o}}\text{C}$, respectively^6,21^.

Depending on the application, PEMFCs stacks can achieve 50% efficiency for ratings between 50 and 500 kW. Moreover, PEMFCs stack has negligible noise, high efficiency (i.e. 50–60%), compact electrolyte that cannot be leaked, long life time, wide power ranges between 1 W and 10 MW and fast startup time^22^. The highest efficiency of PAFCs is equal to 42% which is the lowest of all FCs types, but it is the only FC that has steadily lifetimes of 40,000 h or higher. The efficiency of MCFCs stacks can reach 44%, they can function at temperatures as high as 650 ^o^C, and they don’t require costly heavy metal electrocatalysts that have a 40,000-hour lifespan. Of all FCs, SOFCs stacks have the highest efficiency and lifespan, with an operating temperature of 900 ^o^C. Additionally, because it can only be used in two stages—gas and solid—for the charge transfer reactions at the electrolyte–electrode interface, its design is less complicated than that of PAFCs or MCFC stacks. This simplifies the design by eliminating corrosion and electrolyte management concerns. It can be operated using various types of fuels such as natural, landfill and digester gas as well as biogas and coal syngas^16,23–25^ .

SOFCs stack has a significant role when integrated with solar energy in improving the environmental advantages and reduce the cost of fuel provision^26^. Particular difficulties make it challenging to model, identify, and handle the parameters of SOFCs; further research and effort are needed^1,11^. New theoretical model for SOFC has been developed to control extreme temperature using cooling air bypass^12^. Experimental-based model has been presented to analyze the effect of changing hydrogen and temperature on SOFC’s performance^27^. The performance of two-dimensional SOFCs has been mathematically investigated using various designs and operating conditions^14^. An offline controlling strategy and online parameter identification method have been introduced for SOFCs using particle swarm optimizer (PSO) integrated with the gradient-based search algorithm^28^. The SOFCs stack has been replicated and its components have been modified to increase cell durability and obtain critical information that could not be practically assessed^29^.

There is a significant discrepancy between measured and calculated or anticipated values when using the traditional mathematical approach^30^. Numerous studies have been conducted to identify the optimal SOFC characteristics in order to significantly reduce the cost of SOFCs and improve their performance at lower temperatures using optimization techniques^2,3,6,31,32^. Traditional optimization methods such as linear programming, Newton, and Newton-Raphson could not obtain best answers since they may stick to local optimum solutions^16,19^.

Metaheuristic optimization algorithms were introduced to overcome some of the mentioned drawbacks. It does not require gradient, definite search area information, or convexity of the cost function^33–35^. The bald eagle search optimizer was utilized to obtain SOFC’s precise parameters with good convergence. It showed flexibility with a reasonably significant population size leading to improving the solution process^3^. A recent moth-flame optimizer was proposed to extract SOFC’s parameters as well as cell physical dimensions and properties^4^. An artificial intelligence (AI) method has been developed to determine the information required for assessing and improving the effectiveness and performance of SOFC units^36^. For adjusting the charge transfer coefficients based on the operating conditions and the available datasets, a dedicated approach utilizing artificial neural networks was presented^30^.

Genetic algorithm and its improved versions were used to define SOFC parameters with well control and speed^35^. Differential evolution was applied to find out SOFC’s parameters. The same algorithm was improved and used to realize SOFC’s parameters by including three techniques: restoring crossover rate, selecting the ranking vector, and parameter adjustment^37^. The parameters of the SOFCs were identified using the PSO in conjunction with the cooperative coevolution approach^38,39^. The objective function required to find out SOFC’s parameters were divided to related subfunctions which were solved using hybrid learning integrated with PSO^38^. PSO is simple and has high exploitation behavior but with low exploration capabilities and can untimely converge. The grey wolf optimizer (GWO)^40^was applied to get the SOFC’s parameters because of its simplicity, fast convergence, capability to solve nonlinear and complicated problems. However, it is highly affected by the assumed preliminary population, premature convergence and can stuck in a restricted space^41^. The GWO was improved to avoid the mentioned disadvantages in two updated versions: the chaotic GWO and the adaptive GWO^16^.

Many optimizers were used to identify the SOFC’s parameters such as cooperation search algorithm^2^, bald eagle search optimizer^3^, satin bowerbird optimizer^42^, ranking teaching-learning^17^, artificial bee colony^43^, teaching learning-based optimization methods^17^, interior search algorithm^44,45^and Salp swarm optimizer^46^. The metaheuristic optimizers were also applied for another types of fuel cells. PEMFC’s unknown parameters were identified using many approaches such as biogeography integrated with mutation approach^47^, backtracking search algorithm joined with burger’s chaotic map^48^, improved gorilla troops technique^10^, social learning-based optimizer^49^and feedforward neural network-pelican optimizer^50^.

In this paper, a new optimizer named Chernobyl disaster optimizer (CDO)^51^is employed to define the SOFCs’ unknown parameters. The CDO simulates the process of radiation particles emitted due to the explosion occurred in Chernobyl nuclear reactor. Three major types of particles fly at different speeds from the outburst point with high pressure toward human location point with low pressure area which will harm all human beings. The CDO was tested with optimizing Congress on evolutionary computation 2017 (CEC 2017) test bed suites. Moreover, it was evaluated versus some of reliable optimization techniques, such as sperm swarm optimizer (SSO) and gravitational search algorithm (GSA)^51^. Additionally, the study’s results demonstrated its effectiveness and capabilities.

### Research gaps and inspirations

Based on the previous discussion, the quick survey, and the no-free-launch theorem, it can be concluded that no optimizer can address all difficulties, and there is always opportunity for new optimizers to overcome existing disadvantages and shortages. The latter encouraged numerous researchers to improve optimization methodologies in order to overcome such constraints. The seven SOFCs stack’s parameters are rarely extracted by research studies. The seven obtained parameters were not further verified during dynamic operation. One of the most promising and alternative solutions is to employ techniques such as heuristic-based recent approaches. In addition to that, it can be concluded that each heuristic-based recent approach has its own merits and demerits, and no single algorithm can solve all engineering optimization problems. In addition to that, there is still no solid answer, or it is very difficult to decide that optimization algorithm A is most suitable to optimization problem B of certain characteristics such as degree of non-linearity, convexity, separability of the control variables, modality and etc. Till getting such an answer, attempts shall continue in these endeavors. Notwithstanding numerous strategies as per the above-mentioned to define the uncertain parameters of SOFCs with satisfactory results, and once again, still always there is room for improvements to precisely define the models of SOFCs stacks. In line with this, the authors propose to employ the CDO to identify the undefined parameters of the SOFC’s models.

### Key contributions

The main contributions of this effort may be summarized as follows:

(i) The CDO is utilized to extract the seven parameters of SOFC’s model which compared with another recognized optimizers and proved its dominance.
(ii) The operation of SOFCs with the extracted parameters using the CDO is tested at steady state and dynamic state and its operation is validated.
(iii) Testing of the role of SOFCs with the extracted parameters using the CDO in controlling active and reactive power to network with the support of superconducting magnetic energy storage devices (SMES) and its viability is confirmed.

### Paper structure

The written work of this article begins with this part (Sect. 1); introduction, followed by five more sections. Section 2 presents the problem statement and the steady-state and dynamic mathematical models, while Sect. 3 describes the optimization formulation and complexity analysis. Section 4 describes the proposed CDO’s process and general processes, while Sect. 5 comprises CDO application and verification investigations under steady state and dynamic operating scenarios, followed by concluding remarks.

## Mathematical modeling of SOFCs units and statement

### SOFC operation basics

The SOFC is a solid-state/stationary equipment containing electrolyte of an oxide ion-conducting ceramic material. Hydrogen or carbon monoxide fuels are used as fuels at anode. The oxygen ion O^=^is transferred from cathode to anode via the electrolyte as a negatively charged ion. Water is produced at the anode. The established temperature is sufficient to finish the reaction without an external source. With a long lifespan of 10 to 20 years and a high electrical efficiency of 70 to 75%, this characteristics indicate that SOFCs are one of the best FCs on the market and may be easily implemented in a gas turbine. SOFCs stacks are excellent for distributed generation systems. The main drawback of SOFCs is their high operating temperature, which necessitates the use of materials that are robust^6,52^.

### SOFC steady state modelling

The output voltage of SOFC’s cell can be estimated as given in (1).

1 $$\:{V}_{c}={E}_{oc}-{V}_{tdrop}$$

$\:{V}_{tdrop}$ represents the total voltage drop caused by activation voltage drop $\:{V}_{act}$, concentration voltage drop $\:{V}_{con}$ and ohmic voltage drop $\:{V}_{om}$. $\:{V}_{tdrop}\:$ is expressed as revealed in (2).

2 $$\:{V}_{tdrop\:}={V}_{act}+{V}_{con}+{V}_{om}\:\:$$

The activation potential is an electrochemical potential that is associated with charge transfer due to kinetic interaction. Activation polarization is required to initiate an electrochemical reaction within a fuel cell. The activation voltage could be calculated mathematically as expressed in (3).

3 $$\:{V}_{act}=k\:.\text{ln}\left(\frac{{I}_{den}}{2.{I}_{exden,a}}\right)\:+\:k\:.\text{ln}\left(\frac{{I}_{den}}{2.{I}_{exden,\:c}}\right)$$

The concentration potential is related to changes in concentration of the reactants in the region of electrode which results in a reduction in SOFC’s voltage. Gases partial pressure decreases because of the change in concentrations. This will lead to voltage decrease called concentration voltage that can be expressed as given in (4).

4 $$\:{V}_{con}=-l\:.\text{ln}\left(1-\frac{{I}_{den}}{{I}_{mden}}\right)$$

The ohmic potential is associated with the ionic resistance of the electrolyte material and the internal resistance of the electrodes. The Ohmic overpotential could be expressed as a function of the SOFC’s output current as defined in (5).

5 $$\:{V}_{om}={I}_{out}\:\raisebox{1ex}{${R}_{om}$}\!\left/\:\!\raisebox{-1ex}{${A}_{a}$}\right.$$

The $\:{E}_{oc}$ is assumed equal to Nernst reversible voltage which can be calculated from (6) at real operating state at operating temperature in Kelvin and the oxygen, hydrogen and water pressures; $\:{P}_{O}$, $\:{P}_{H}\:,\:{P}_{W},$respectively^53,54^.

6 $$\:{E}_{oc}=\:{E}_{s}+\:\frac{R.T}{2{D}_{f}}\:.\text{l}\text{n}\left(\frac{{P}_{H}\sqrt{{P}_{O}}}{{P}_{W}}\right)$$

If fuel cell stack has number of identical cells $\:{N}_{c}$ connected in series, the total system voltage $\:{V}_{out}$can be calculated as given in (7)^55^; assuming that all cells behave the same. The reversible potential is the maximum voltage that an ideal cell could deliver under specific operating conditions.

7 $$\:{V}_{out}=\:{V}_{C}{\:.N}_{c}\:$$

### SOFC’s dynamic modelling

The SOFC’s operation and the molar flow are substantially affected by the pressure variations of hydrogen, air, and water relating to time^44,52^. $\:{E}_{oc}$ can be reformulated as a function of $\:\varDelta\:{B}_{t}$, the operating temperature $\:\text{T}$, $\:{P}_{H}$, $\:{P}_{O}$ and $\:{P}_{W}\:$as per (8)^44,56^.

8 $$\:{E}_{oc}=-\left\{\raisebox{1ex}{$\varDelta\:{B}_{t}$}\!\left/\:\!\raisebox{-1ex}{$2{D}_{f}$}\right.\right\}+\left\{\raisebox{1ex}{$R.\:T$}\!\left/\:\!\raisebox{-1ex}{$2{D}_{f}$}\right.\right\}.\:ln\left\{\raisebox{1ex}{${P}_{H}.\sqrt{{P}_{O}}$}\!\left/\:\!\raisebox{-1ex}{${P}_{W}$}\right.\right\}$$

The hydrogen and oxygen molar flow $\:{H}_{F}$ and $\:{O}_{F},$respectively can be estimated as per (9) and (10), respectively^57^:

9 $$\:{H}_{F}={P}_{H}\:\left\{\raisebox{1ex}{${C}_{a}$}\!\left/\:\!\raisebox{-1ex}{$\sqrt{{H}_{M}}$}\right.\right\}=\:{P}_{H}{.C}_{H}$$

10 $$\:{O}_{F}={P}_{O}\:\left\{\raisebox{1ex}{${C}_{a}$}\!\left/\:\!\raisebox{-1ex}{$\sqrt{{O}_{M}}$}\right.\right\}=\:{O}_{P}{.C}_{O}$$

The oxygen and hydrogen molar constants are represented by $\:{C}_{O}$ and $\:{C}_{H}.$ The change of pressure with respect to time is expressed in (11).

11 $$\:\raisebox{1ex}{$d{P}_{H}$}\!\left/\:\!\raisebox{-1ex}{$dt$}\right.=\:\left(B.\frac{{T}_{op}}{{A}_{v}}\right)\:\left\{{F}_{{IN-H}_{2}}-{F}_{{OU-H}_{2}}-{F}_{{RH}_{2}}\right\}$$

$\:{F}_{{RH}_{2}}$ is the real combined hydrogen which can be evaluated as per (12).

12 $$\:{F}_{{RH}_{2}}=N.\:\frac{{I}_{out}}{2{D}_{f}}\:$$

The combined hydrogen constant $\:{K}_{R}\:$can be calculated as (13).

13 $$\:{K}_{R}=\:\frac{N}{4{D}_{f}}$$

Then, $\:{F}_{{RH}_{2}}$ can be re-expressed as per (14).

14 $$F_{RH_2}= 2\, K_R{\cdot} I_{out}$$

SOFC’s dynamic performance is highly affected by controlling $\:{P}_{H}$, $\:{P}_{O},$ and $\:{W}_{p}.$ These three variables can be rewritten in S-domain as defined in (15)-(17).

15 $$\:{P}_{H}\left(S\right)=\frac{1/{C}_{{H}_{2}}}{1+{\tau\:}_{{H}_{2}}}\:\left\{{F}_{{IN-H}_{2}}-2{K}_{R}.{I}_{out}\right\}$$

16 $$\:{P}_{O}\left(S\right)=\frac{1/{C}_{{O}_{2}}}{1+{\tau\:}_{{O}_{2}}}\:\left\{{F}_{{IN-O}_{2}}-2{K}_{R}.\:{I}_{out}\right\}$$

17 $$\:{W}_{p}\left(S\right)=\frac{1/{C}_{{H}_{2}O}}{1+{\tau\:}_{{H}_{2}O}}\:2{K}_{R}.\:{I}_{out}$$

The flow rate of fuel $\:{F}_{f}$, hydrogen $\:{\:F}_{{H}_{2}}$, and $\:{F}_{{O}_{2}}$can be expressed correlated to SOFC’s total current as in (18)-(20)^44,56^.

18 $$\:{F}_{f}=\frac{2{K}_{R}.\:{I}_{out}}{{S}_{f}}$$

19 $$\:{F}_{{H}_{2}}\left(s\right)=\frac{1}{1+{\tau\:}_{f}s}{F}_{f}$$

20 $$\:{F}_{{O}_{2}}\left(s\right)=\frac{{F}_{{H}_{2}}}{{R}_{{T}_{H-O}}}$$

Once again, many $\:{N}_{c}$ cells of SOFCs are connected to form a stack to raise its rating with output voltage $\:{V}_{st}$ as specified in (21), assuming that all cells behave with same performance.

21 $$\begin{aligned}\:{V}{st}&={N}{c}.{V}{c}\\ &={N}{c}.\left\{-\raisebox{1ex}{$\varDelta\:{B}{t}$}\!\left/\:\!\raisebox{-1ex}{$2{D}{f}$}\right.+\left({G}{u}.\frac{{T}{op}}{2{D}{f}}\right).\:ln\left\{\raisebox{1ex}{${P}{H}.\sqrt{{P}{O}}$}\!\left/\:\!\raisebox{-1ex}{${P}{W}$}\right.\right\}\right. \\&\quad\left.-\left(k\:.\text{ln}\left(\frac{{I}{den}}{2.{I}{exden,a}}\right)\:+\:k\:.\text{ln}\left(\frac{{I}{den}}{2.{I}{exden,\:c}}\right)-l\:.\text{ln}\left(1-\frac{{I}{den}}{{I}{mden}}\right)+{I}{out}\:{R}{om}\right)\right\}\end{aligned}$$

## Proposed optimization and problem formulation

The CDO is utilized to extract the seven unknown parameters of SOFCs such as $\:{E}_{s}$,$\:{\:I}_{exden,a}$, $\:{I}_{exden,c}$, $\:{I}_{mden}$, *k*,* l* and $\:{R}_{om}$. The aim of the fitness function, $\:FF$ is to minimize the difference between mean square error summation among the estimated voltage; $\:{V}_{out}$ and the real measured output voltage; $\:{V}_{m}$for number of tests (N) as revealed in (22) under the some operating and design constraints given in (23)^16,17,19,42,43^.

22 $$\:FF=Minimize\left(\sum\:_{i=1}^{N}{({V}_{out,i}-{V}_{m,i})}^{2}\right)$$

23 $$Subject to: \:\left\{\begin{array}{c}{{E}_{s}}^{min}\:\le\:{E}_{s}\le\:{{E}_{s}}^{max}\:\\\:{{I}_{exden,a}}^{min}\:\le\:{I}_{exden,a}\le\:\:{{I}_{exden,a}}^{max}\\\:{{I}_{exden,c}}^{min}\:\le\:{I}_{exden,c}\le\:\:{{I}_{exden,c}}^{max}\\\:\:{{I}_{mden}}^{min}\:\le\:{I}_{mden}\le\:\:{{I}_{mden}}^{max}\\\:{I}_{mden}>{I}_{exden}\\\:{k}^{min}\:\le\:\:k\le\:\:{k}^{max}\\\:\:\:{l}^{min}\:\le\:l\le\:\:{l}^{max}\\\:{{R}_{om}}^{min}\le\:{R}_{om}\:\le\:\:{{R}_{om}}^{max}\end{array}\right.$$

## Procedures of Chernobyl disaster optimizer

CDO is one of the most newly meta-heuristics algorithms published in February 2023 by Shehadeh^51^. It is categorized as one of the physical-based category that imitates the worst nuclear accident in history (Chernobyl disaster). The Chernobyl accident occurred in 1986, when pressure and temperature rising inside reactor No. 4 caused an explosion. This explosion resulted in three radiations of varying speeds and masses. There are three radiations: alpha, beta, and gamma. Alpha radiation is the heaviest and biggest, with a low speed since it contains two protons and two neutrons. Alpha particles are positively charged and intensely ionizing. Beta particles have a negative charge, a very little mass, and are moderately ionizing at high speeds. Gamma particles are defined as electromagnetic radiation with a high frequency and short wavelength, minimal mass, and weak ionization at high speeds. The aforementioned particles are dangerous to humans because they go from the explosion location to the human region and attack them. This calamity was recreated using an algorithm, which will be exhibited now.

The gradient descent factor $\:\nabla\:$ of alpha, beta and gamma while attacking human can be calculated as given in (24).

24 $$\:\nabla\:=\text{c}(X\left(t\right)-\rho\:.\varDelta\:)$$

c is a constant and is equal to 0.25 for 𝛼 particles, 0.5 for 𝛽 particles and 1 for γ particles. $\:\rho\:$ can be calculated as in (25).

25 $$\:\rho\:=\frac{{A}_{h}}{c.log\left(rand\left(1:v\right)\right)}-\left(W.rand\left(\dots\:\:\right)\right)$$

$\:W$ is calculated as stated in (26).

26 $$W=3-1</span>\text{*}\frac{3}{max\_Iter}$$

The difference between particles position and human position $\:{\Delta\:}$ can be calculated as specified in (27).

27 $$\:\varDelta\:=\left|{A}_{p}.X\left(t\right)-{X}_{T}\left(t\right)\right|$$

$\:{X}_{T}\left(t\right)$ can be calculated according to the Galileo Galilei motion equations as arranged in (28).

28 $$\:{X}_{T}\left(t\right)=\frac{{\nabla\:}_{{\upalpha\:}}.{\nabla\:}_{{\upbeta\:}}.{\nabla\:}_{{\upgamma\:}}}{3}$$

**Fig. 1:**
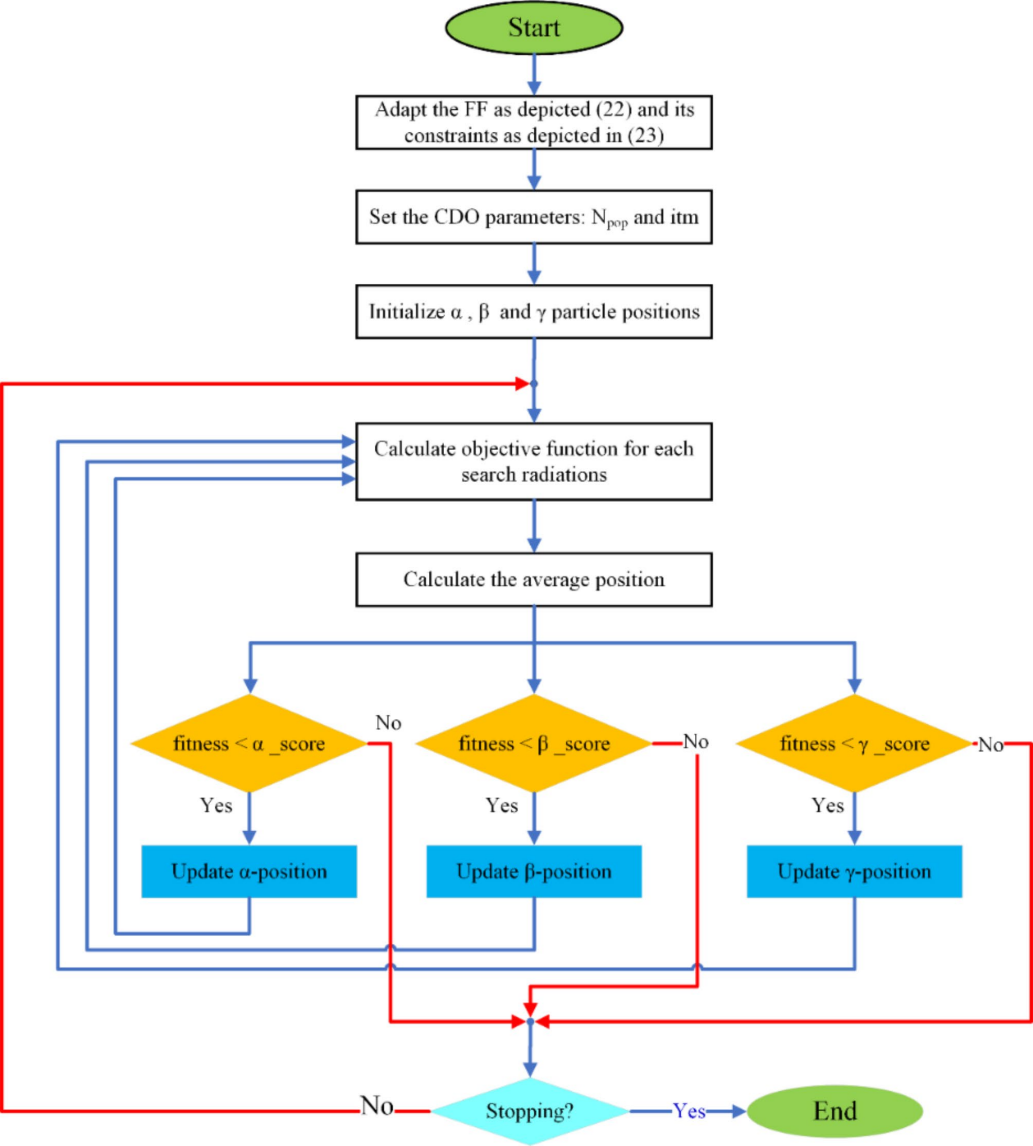
General procedures of the employed CDO.

The flowchart in Fig. 1 depicts the CDO’s general procedures. According to the developer of the CDO’s original effort, the effectiveness of the CDO is assessed (quantitative and qualitative) using 23 mathematical optimization problems chosen from CEC 2017 to compare against the SSO and GSA. The CDO exploration beat both the SSO and the GSA, particularly for noisy and difficult issues, and the CDO has a higher speed rate than these algorithms.

The time complexity of the CDO is measured using Big-O as $\:O\left({N}_{pop}.m.\text{m}\text{a}\text{x}\_Iter\right);$ where $\:{N}_{pop}$ is the population size and $\:m$ defines the problem dimension. The summarized pseudo code for the CDO is described as follows:

**Table:** 

Pseudo code of the CDO Algorithm
Begin 1: Initialize $\:\alpha\:$, β, and γ particle positions
2: Initialize the positions of search radiations
3: While ($$\:t<=\text{m}\text{a}\text{x}\_Iter$$)
4: Estimate the fitness of all agents ($\:F\left(i\right),\:i\:=\:1:{N}_{pop}$)
5: If fitness < α_score
6: Update α-position
7: End if
8: If fitness < β_score
9: Update β-position
10: End if
11: If fitness < γ_score
12: Update γ-position
13: End if
14: For all particles α, β, and γ, update the position on XY plan.
15: Calculate gradient descent factor for α, β, and γ particles using (24)
16: Update average total position using (28)
17: t = t + 1
18: End While
19: Return min (Fitness value)

## Case studies and validation of CDO

To evaluate the effectiveness of the CDO algorithm, it is applied to a tubular SOFCs stack with the data given in^35^ to define the best values of the unknown parameters of SOFC’s model. The performance of the SOFCs stack is assessed under both steady-state and dynamic conditions. At steady-state, the SOFCs unit is gradually loaded and its performance is recognized. Furthermore, SOFCs unit’s performance is analyzed when connected to direct load, main network and SMES to control power supplied to the network with sudden changes for dynamic testing.

### Applications of CDO at steady-state

The CDO algorithm is established and verified under MATLAB environment installed on an Intel(R) Core (TM) i7-4710HQ LAPTOP with CPU @ 2.5GHZ and 8 GB RAM for the mentioned SOFCs stack at $\:{800}^{o}C$ and $\:{900}^{o}C$. The obtained results are compared with another trusted published optimizers. Lower and upper limits of the controlled parameters are decided based on specifications of SOFCs unit which depends on field equipment ratings. Final selections of lower and upper bounds of the tunable parameters are conducted on the basis of exhaustive simulation studies and in line the recent literature for fair comparisons (see Table 1). The final best values of the controlled tunable parameters are decided when minimum value of the FF is realized and feasible solution within the predefined range of the limits are obeyed.

The CDO supervises this process, which eventually produces the final optimal values for the specified parameters. Furthermore, the best characteristics of the CDO algorithm, including the maximum number of iterations and population size, have been determined by trial and error and rely on the designer’s experience. This criterion is the most commonly used in many meta-heuristic’s optimization algorithms. The final adapted control parameters of the CDO are: number of particles is equal to 50 with maximum iterations of 400 iteration. Table 2 shows the appraised parameters using the CDO-based methodology at $\:{800}^{o}C$ (Column 6) and $\:{900}^{o}C$ (Column 11). Table 2 arranges a comparison between the CDO and recent four challenging optimizers at $\:{800}^{\text{o}}\text{C}$ and $\:{900}^{\text{o}}\text{C}$ available in the literature. It is evident that given the two situations under study, the CDO was able to achieve the lowest SMSE out of all optimizers.

**Table 1:** Calculated parameters of the cylindrical SOFC using the CDO at $\:{800}^{o}C$ and $\:{900}^{o}C$.

Parameters	$\:{\varvec{E}}_{\varvec{s}}$ (V)	k (V)	I_exden,a_(A/cm^2^)	I_exden,c_(A/cm^2^)	l (V)	I_mden_(A/cm^2^)	R_om_(kΩ.cm^2^)	SMSE ($\:\varvec{\mu\:}{\varvec{V}}^{2}$)	Time (s)
Parameters’ Limits	0.8 ~ 1.2	0 ~ 1	0 ~ 100	0 ~ 100	0 ~ 1	0 ~ 1000	0 ~ 1	-	-
Estimated Parameters at $\:{800}^{o}C$	0.9270	0.7530	0.0343	0.983	0.2230	0.0635	0.652	3.46	171.524
Estimated Parameters at $\:{900}^{o}C$	0.8859	0.2075	0.1224	0.372	0.7698	0.1731	0.165	7.38	122.300

**Table 2:** Calculated parameters of the cylindrical SOFC using the CDO against various optimizers.

Parameter	$\:{800}^{\varvec{o}}\varvec{C}$					$\:{900}^{\varvec{o}}\varvec{C}$				
	COA^3^	MPA^57^	MFA^58^	SCA^59^	CDO	COA^3^	MPA^57^	MFA^58^	SCA^59^	CDO
$\:{E}_{s}$ (V)	1.1196	1.1196	1.1196	1.1196	0.927	1.1104	1.1105	1.1164	1.1158	0.8859
*k* (V)	0.0575	0.0624	0.0545	0.0591	0.753	0.0380	0.0717	0.0265	0.0577	0.2075
$\:{I}_{exden,a}$ (A/cm^2^)	16.5273	17.2821	16.000	16.000	0.0343	29.4774	26.0306	26.0000	26.0000	0.1224
$\:{I}_{exden,c}$ (A/cm^2^)	6.1477	6.7761	5.6407	6.3395	0.983	6.9722	6.6919	4.1032	4.0000	0.372
*l* (V)	0.0462	0.0552	0.0375	0.0354	0.223	0.0663	0.0641	0.0588	0.0943	0.7698
$\:{I}_{mden}$ (A/cm^2^)	154.4925	154.9336	149.3598	157.000	0.0635	159.9422	160.000	159.6766	160.000	0.1731
$\:{R}_{om}$ (KΩ.cm^2^)	0.0062	0.0060	0.0064	0.0062	0.652	0.0040	0.0058	0.0043	0.0058	0.1650
SMSE ($\:{V}^{2}$)	54.68e-6	4.911e-4	7.549e-4	9.815e-2	3.46e-6	2.413e-4	1.228e-5	1.224e-2	1.726e-1	7.38e-6

**Fig. 2:**
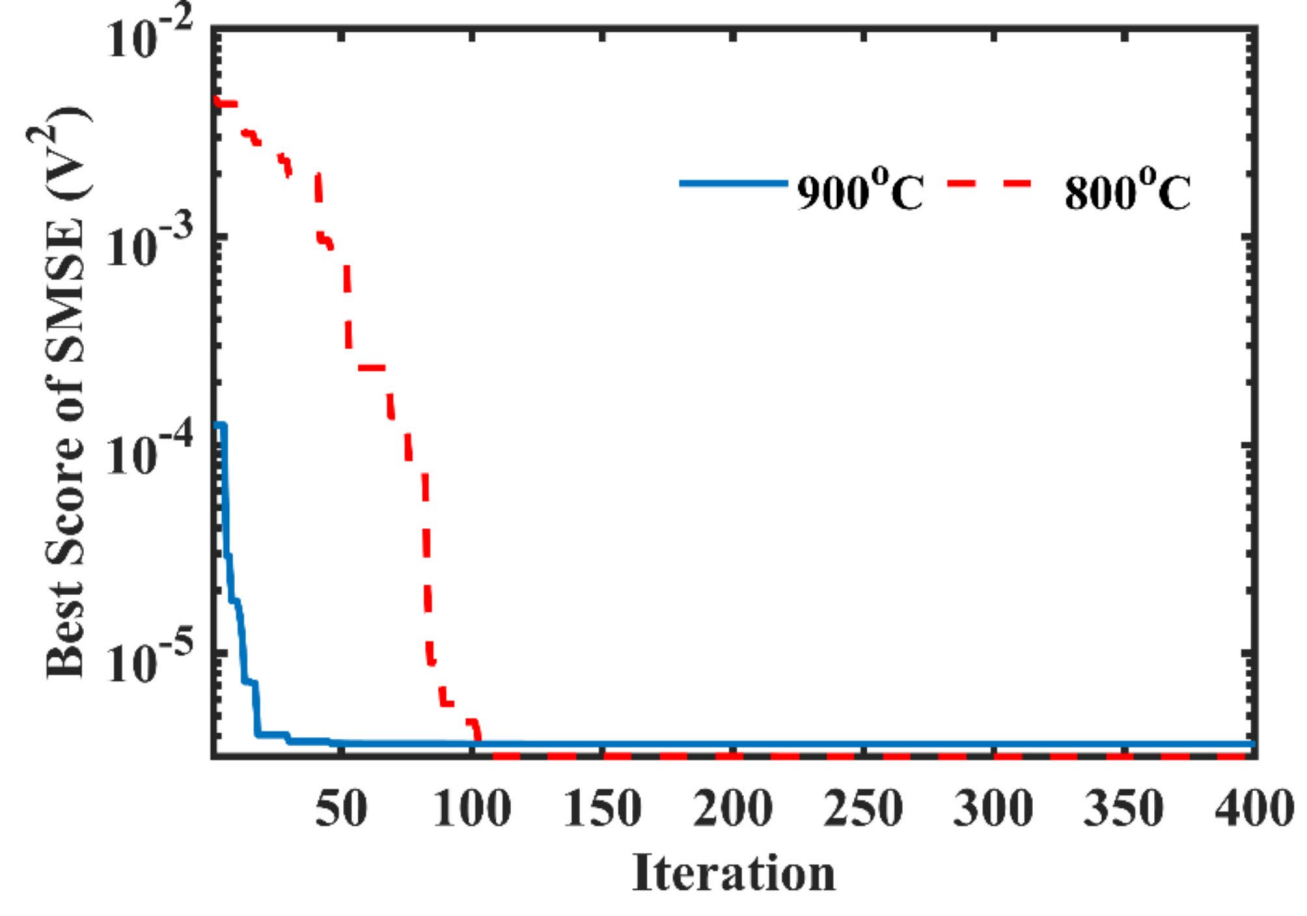
Convergence trends of the CDO at $\:{800}^{\text{o}}\text{C}$ and $\:{900}^{\text{o}}\text{C}$.

To verify the reliability and stability of the CDO algorithm, the convergence patterns are given in Fig. 2. It can be noticed that it starts to be steady from 150th iteration at $\:{800}^{o}C$ and 50th iteration at $\:{900}^{o}C$.

**Fig. 3:**
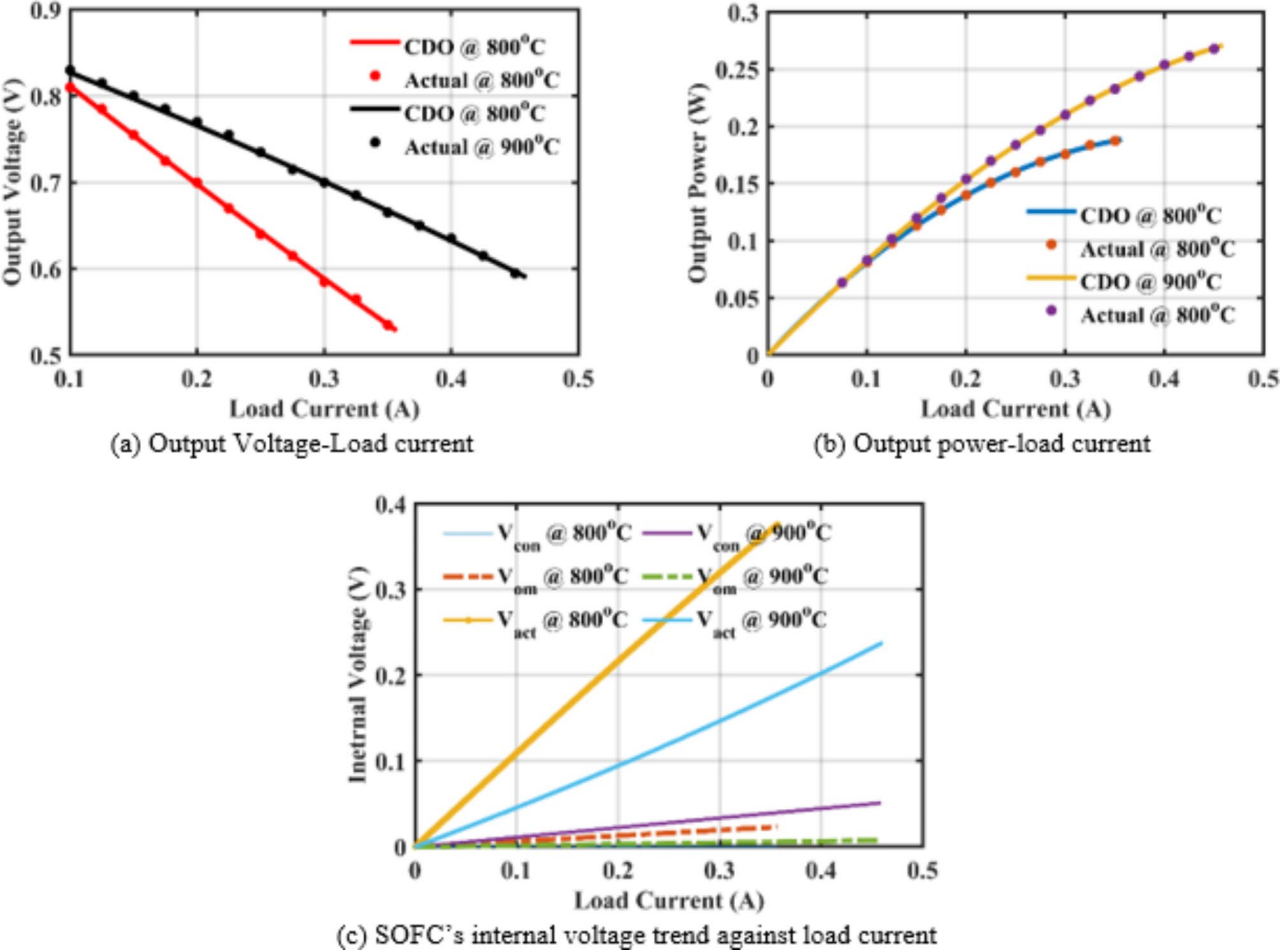
Comparison of the SOFC behavior when using the CDO results and the actual values with changing load at $\:{800}^{o}C$ and $\:{900}^{o}C$.

For further verification, the CDO’s performance is compared to the actual values of the SOFCs unit, as shown in Fig. 3(a)-(b). Figure 3(a)-(c) shows the output voltage, output power, and internal voltage variations, respectively at $\:{800}^{o}C$ and $\:{900}^{o}C$.

### Dynamic performance of SOFC Integrated with SMES

**Fig. 4:**
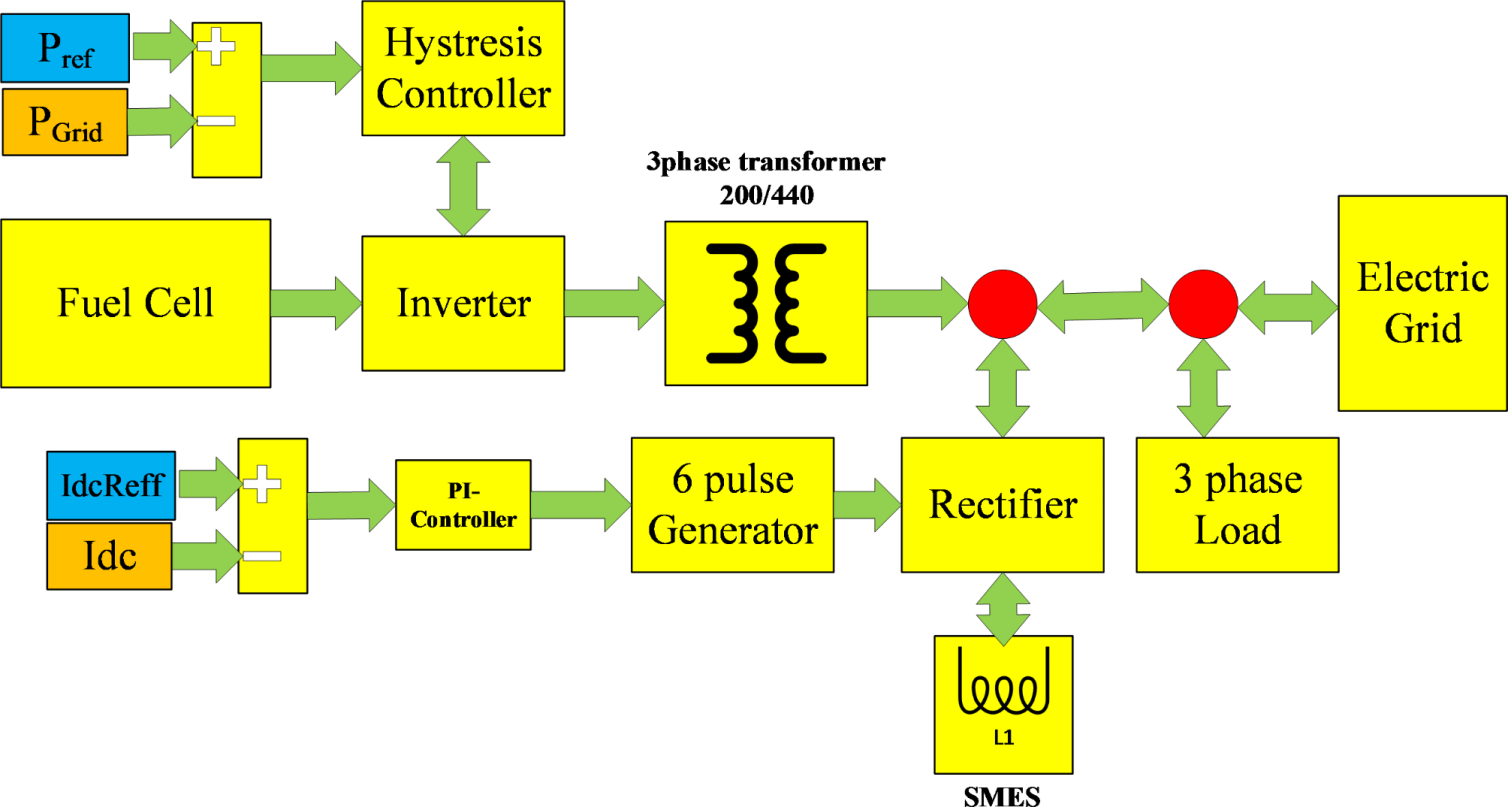
Overall system for SOFCs stack with the controlled proposed power system application.

To increase satisfaction with CDO’s ability in identifying SOFC’s parameters, the SOFC’s dynamic behavior is investigated and confirmed. A test model is built up using MATLAB/Simulink tool which includes a SOFCs stack connected to an electric power grid, integrated with SMES, direct load, and two controllers, as shown in Fig. 4. The role of SOFCs stack together with SMES in managing active and reactive power transmitted to the grid is investigated. The SOFCs stack composed of 384 cells connected in series and can deliver 100 kW with data shown in Table 3^6,42,56,60,61^.

**Table 3:** Dynamic model parameters of the 100 kW SOFCs stack.

Parameter	Value	Parameter	Value
$\:\text{T}$ (^o^C)	1273	Rated power (kW)	100.0
$\:{N}_{c}$	384	$\:{r}_{\text{H}-O}$ (s)	1.145
$\:{K}_{r}\:$	0.996e-6	$\:{\tau\:}_{f}$ (s)	5
$\:{K}_{{H}_{2}}$	84.3e-5	$\:{\tau\:}_{{H}_{2}}\:$(s)	26.1
$\:{K}_{{H}_{2}O}$	28.1e-5	$\:{\tau\:}_{{H}_{2}O}\:$(s)	78.3
$\:{K}_{{O}_{2}}$	252e-5	$\:{\tau\:}_{{O}_{2}}$ (s)	2.91
$\:{U}_{{O}_{\text{p}\text{t}}},\:\text{F}\text{u}\text{e}\text{l}\:\text{U}\text{t}\text{i}\text{l}\text{i}\text{z}\text{a}\text{t}\text{i}\text{o}\text{n}\:\text{f}\text{a}\text{c}\text{t}\text{o}\text{r}$	0.85	$\:{\tau\:}_{r}\:$(s)	0.8

$$\:{K}_{r}$$ is a constant = $$\:N$$/4F measured in kmol/(A.s), $$\:{K}_{{H}_{2}}$$, $$\:{K}_{{H}_{2}O}$$, and $$\:{K}_{{O}_{2}}$$ are the gain constant values of $$\:{H}_{2}$$, $$\:{\:H}_{2}O$$ and $$\:\:{O}_{2}$$ in kmol/(atm.s) respectively, $$\:{U}_{{O}_{\text{p}\text{t}}}$$is the utilization factor, $$\:{\tau\:}_{f},$$$$\:{\tau\:}_{{H}_{2}}$$, $$\:{\tau\:}_{{H}_{2}O}$$, $$\:{\tau\:}_{{O}_{2}}$$ and $$\:{\tau\:}_{r}$$ are time constants of fuel process, $$\:{H}_{2}$$, $$\:{\:H}_{2}O$$ and $$\:\:{O}_{2}$$ and electrical responses in seconds respectively.

#### Description and modelling of the test system

The system is composed of SOFC connected to IGBT inverter with three phase output, 200 V, 60 Hz to be transformed to 440 V connected to the electric network and three phase 10 kW load as depicted in Fig. 4. The system has two controllers, the first one is used to control the inverter to control SOFCs output and the other one is to control the SMES. The inverter uses hysteresis controller to control the active power by employing direct-axis current while holding reactive power near to zero VArs. The proposed SMES control system always uses DC/DC bidirectional converter to achieve the different control states. Equations (29) and (30) represent the modelling of $\:{P}_{SMES}$ and $\:{E}_{SMES}$^62,63^.

29 $$\:{E}_{SMES}=\frac{1}{2}{L}_{SMES}{I}_{SMES}^{2}$$

30 $$\:{P}_{SMES}=\frac{d{E}_{SMES}}{dt}={L}_{SMES}.{I}_{SMES}.\frac{d{I}_{SMES}}{dt}={V}_{SMES}.{I}_{SMES}$$

The SMES control system uses PI-controller. The difference between $\:{I}_{ref}$ and $\:{I}_{SMESDC}$ is delivered to the used PI-controller to eliminate the steady-state error from the input signals. The controller’s output is passed to the six-pulse generator to create the required control signals gates which are responsible to decide the SMES’s proper operation mode (charging, discharging, or storing mode). In the proposed power system, the $\:{P}_{SOFC}\:$should satisfy power balance as given in (31)^64^.

31 $$\:{P}_{SOFC}={P}_{load}\pm\:{P}_{SMES}\pm\:{P}_{Grid}$$

The test system feeds SMES with value of inductance $\:{L}_{SMES}$ = 1.8 H that can be charged, discharged, or stored as per the decided mode. The integral constants gain values of the proposed SMES controller are assumed 65 and 5000, respectively (Obtained by trials and errors procedure). The objective of the controlling process is to track the coil current and to generate the required six-pulse to feed the SMES rectifier circuit. Taking 50 kW as base value, the simulation is started at t = 0 by considering the network requires active power with 0.3 pu ($\:{P}_{ref}$ = 0.3 pu.) and increased to 1 pu at t = 0.4 s. The dynamic performance of the system is observed and analyzed in the following subsection.

#### Application results and discussions

**Fig. 5:**
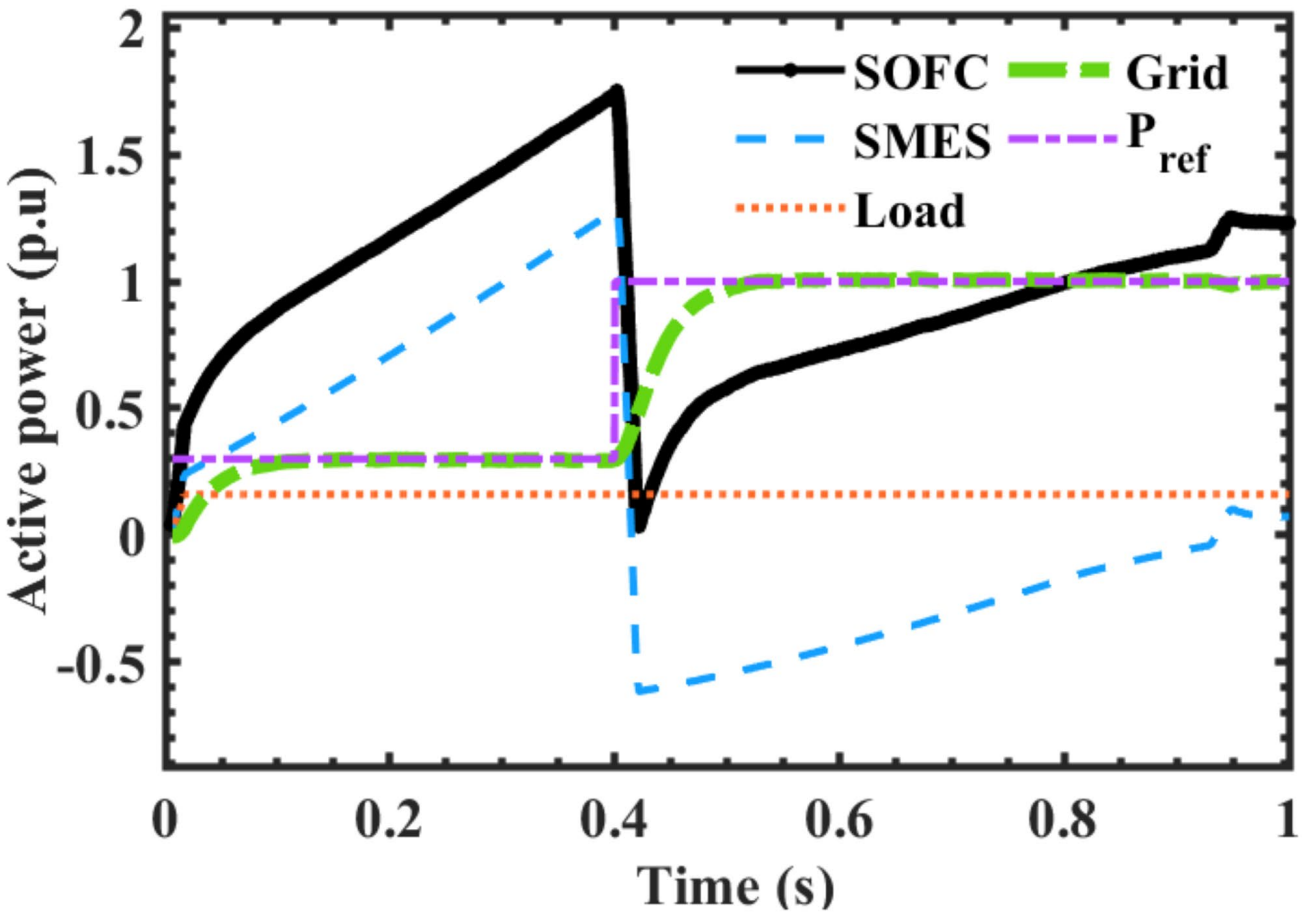
Responses of system elements according to the variation of P_ref_ of the main controller.

**Fig. 6:**
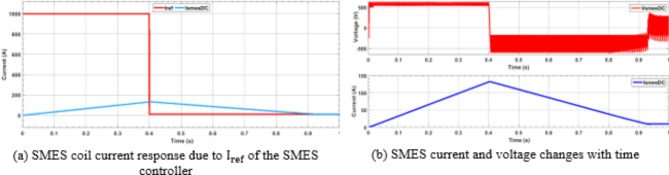
SMES current and voltage behavior due to changing in reference current.

**Fig. 7:**
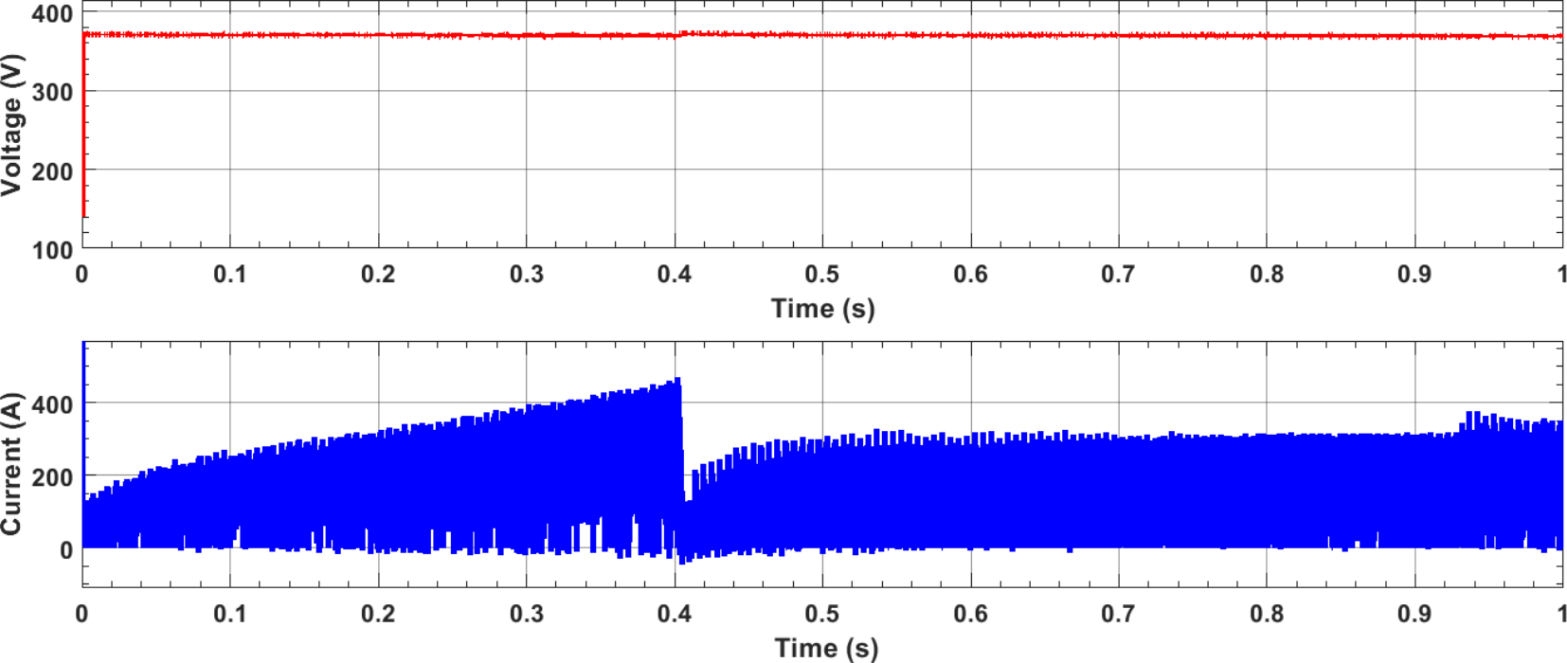
SOFCs unit’s current and voltage (DC value).

**Fig. 8:**
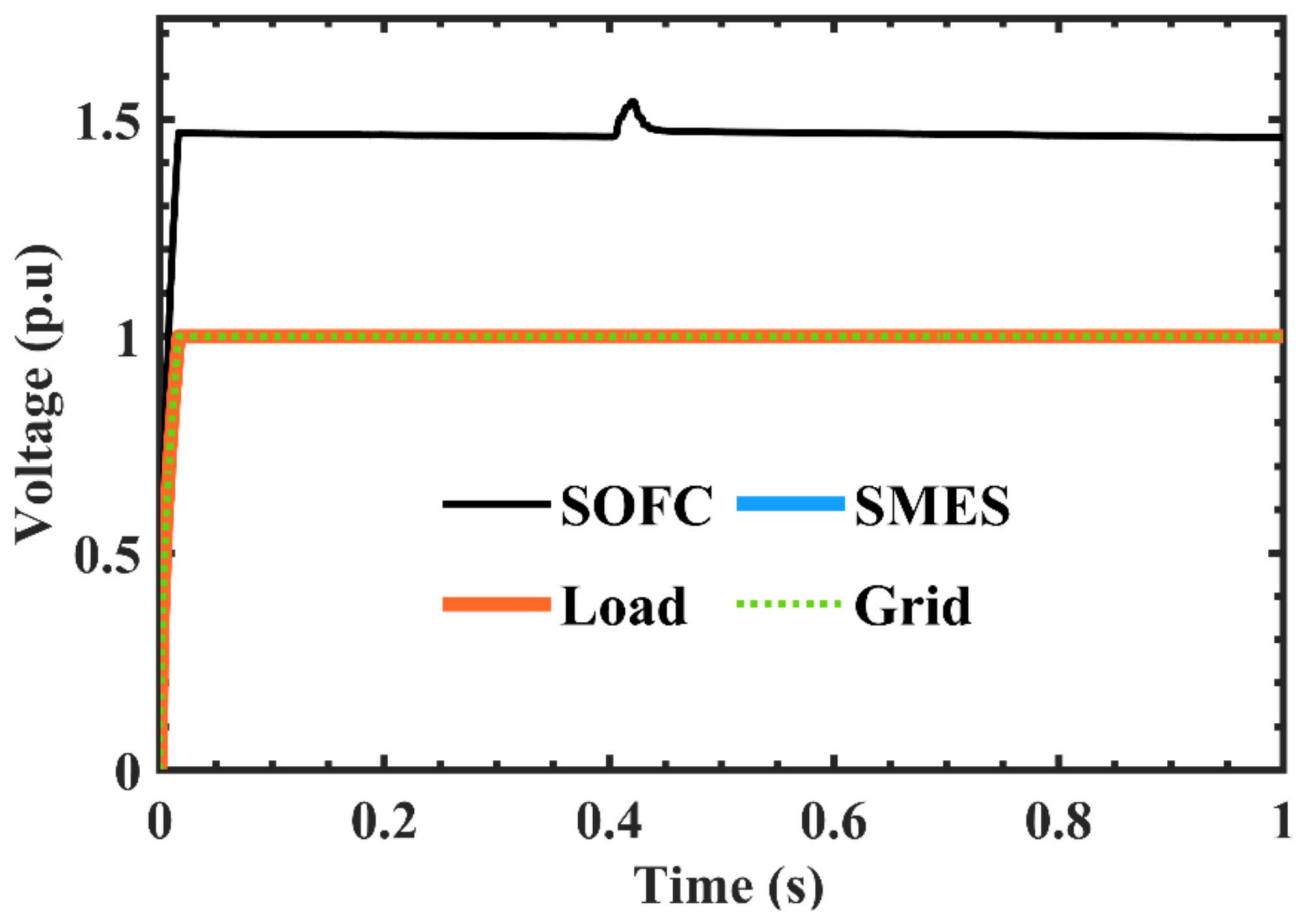
Variations of terminal voltage for SOFCs unit, SMES, load, and the grid.

**Fig. 9:**
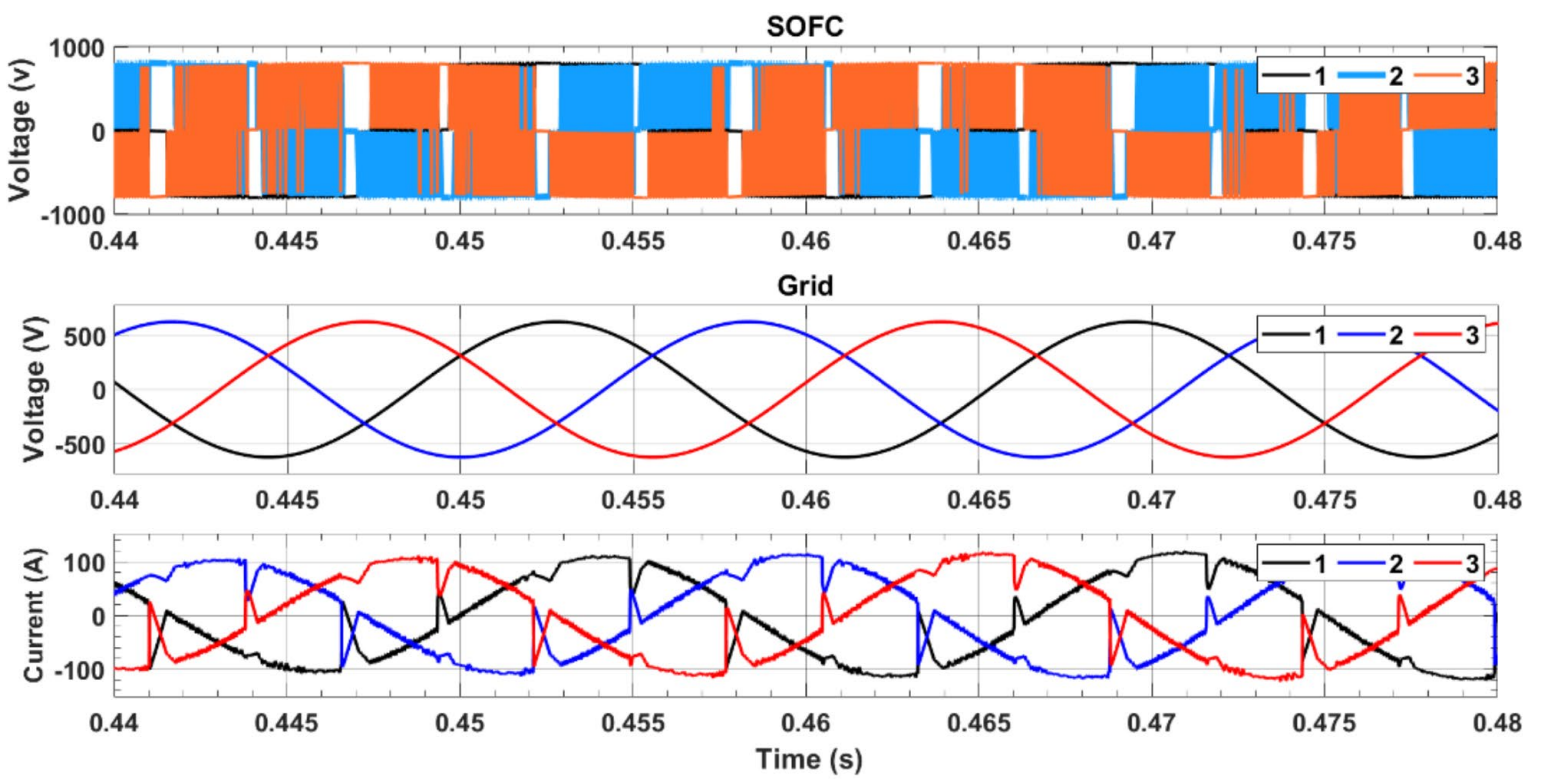
Variation of instantaneous voltage for SOFCs unit, and the grid.

**Fig. 10:**
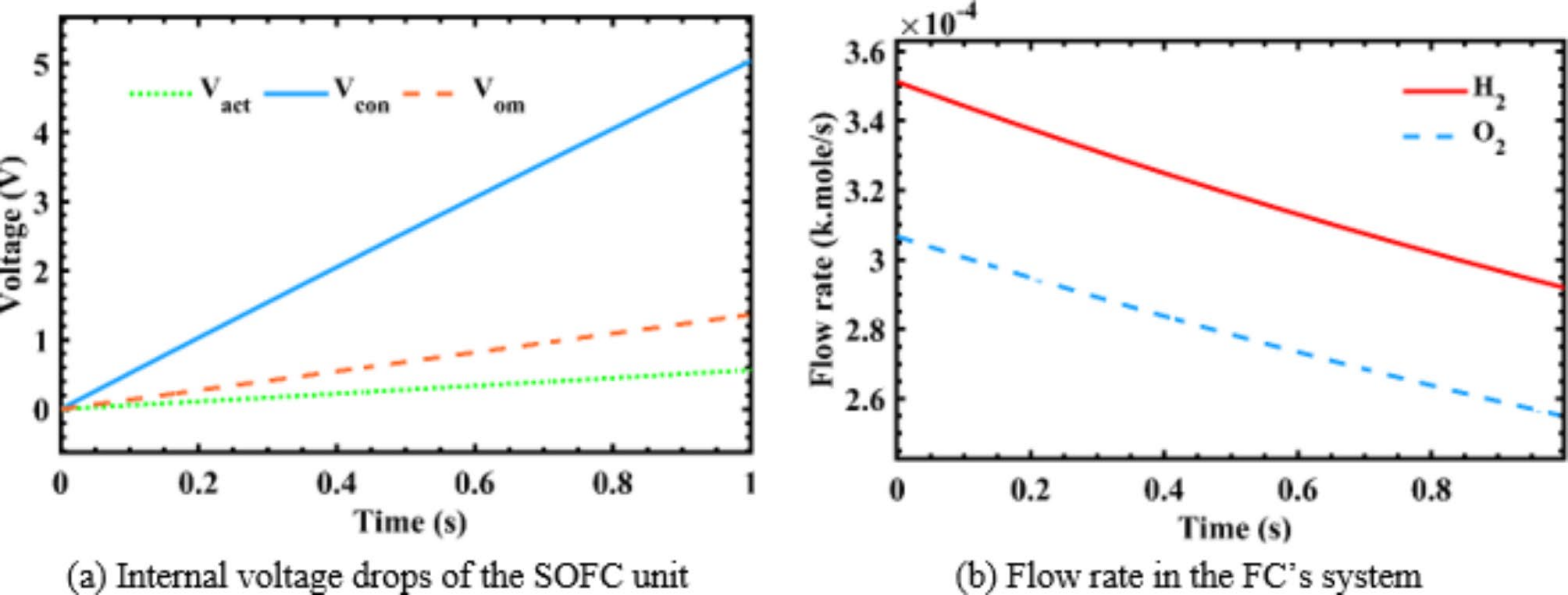
SOFC’s internal voltage drops and flow rates behavior with time.

The behavior of SFOCs stack, SMES, load and grid according to the changing at t = 0 s and t = 0.4 s are depicted in Figs. 5, 6, 7, 8, 9 and 10. The variations of active power for all system elements are characterized in Fig. 5. It can be observed that $\:{P}_{SOFC}$ equal to the power required by the grid, load and SMES at any time of the simulation; achieving power balance. The period from t = 0 s to t = 0.4 s, SOFCs unit charges the SMES while supplying network and load requirements. At t = 0.4 s when the required $\:{P}_{Grid}$ is suddenly increased more than 3 times existing load from 0.3 pu to 1 pu, the SOFC’s output is instantly dropped while the SMES is discharged to compensate the drop of fuel cell and fulfill the system requirements. SOFC’s output starts to gradually increase again within 0.1 s to recover the new load by increasing its fuel in addition to the SMES discharged energy up to t = 0.98 s. The fuel cell is forced to fix the active power fed to the grid near by the reference signal $\:{P}_{ref}\:$by the fuel cell controller. At t = 0.98 s, SOFC becomes capable of supplying the grid, load and storing energy in the SMES (storage mode) up to the end of simulation at t = 1 s.

Figures 6 and 7 depict the variations in the SMES and SOFCs units’ current and voltage over the period of the simulation. Figure 8 shows the terminal RMS voltage for the grid, load, SMES, and SOFCs unit. Figure 9 displays the SOFCs unit and grid’s instantaneous voltage and current.

The behavior of SOFC’s output voltage and internal voltage drops are illustrated in Fig. 10(a). Besides, the associated $\:{\text{H}}_{2}$ and $\:{\text{O}}_{2}$ flow rates behaviors are shown in Fig. 10(b).

Notably, the SOFCs stack can charge the SMES and supply network and load with reliability. Additionally, as previously mentioned, the impact of changing the load on SOFCs stack has been smoothly managed within 0.1 s. The SMES can maintain network functionality when SOFCs stack fails due to a sudden increase in demand.

## Conclusions

A recent CDO has been applied to extract best seven unknown parameters of SOFCs units. Steady-state and dynamic behaviors of the SOFCs stack have been investigated and analyzed. The results are compared in contrast to well-known challenging optimizers at various operating states. The CDO could produce the best minimum sum of mean square error among all optimizers such as 3.46 µV^2^ at $\:{800}^{o}C$ and 7.38 µV^2^ at $\:{900}^{o}C$ were cropped. The dynamic operation of a SOFCs unit was investigated by connecting it to a network, load, and SMES equipped with two controllers that controlled active and reactive power fed into the network. One of the controllers is used to operate the inverter between the SOFCs stack and the network, while the other controls the SMES. The SOFCs stack could provide reliable network and load power while also charging the SMES. Furthermore, the effect of adjusting the load on the SOFCs unit has been smoothly managed within 0.1 s. The SMES was able to maintain the required active power to the network when SOFC units suddenly failed due to a sudden load rush. SOFC stack demonstrates its potential to charge and even store SMES in along with its normal load. The dynamic response of internal voltage drops, flow rate changes over time, and modification of SOFC instantaneous voltage all contribute to the success of the CDO-based methodology.

## Data Availability

No, I do not have any research data outside the submitted manuscript file.

## Abbreviations

DER: Distributed energy resources
FCs: Fuel cells
SOFCs: Solid oxide FCs
PEMFCs: Proton exchange membrane FCs
PAFCs: Phosphoric acid FCs
MCFCs: Molten carbonate FCs
CDO: Chernobyl disaster optimizer
GSA: Gravitational search algorithm
SSO: Sperm swarm optimizer
SMES: Superconducting magnetic energy storage
COA: Coyote optimization algorithm
MFA: Moth flame Algorithm
MPA: Marine predator algorithm
SCA: Sine cosine algorithm
GWO: Grey wolf optimizer
PSO: Particle swarm optimizer
CEC: Congress on evolutionary computation
GB: Giga Byte
RAM: Random access memory
CPU: Central processing unit
IGBT: Insulated gate bipolar transistor

## List of symbols

$\:{V}_{C}$: Output voltage of SOFC, V
$\:{E}_{oc}$: Open circuit voltage of SOFC, V
$\:{V}_{act}$: Activation voltage drop, V
$\:{V}_{tdrop}$: Total voltage drops, V
$\:{V}_{con}$: Concentration voltage drop, V
$\:{V}_{om}$: Ohmic voltage drop, V
$\:{I}_{den}$: Load current density, mA/cm^2^
$\:{I}_{exden,a}$: Anode exchange current density, mA/cm^2^
$\:{I}_{exden,c}$: Cathode exchange current density, mA/cm^2^
$$\:{I}_{mden}$$: Maximum of $$\:{I}_{den}$$
$$\:l$$: Constant, V
$\:{I}_{out}$: SOFC output current, V
$\:{R}_{om}$: Ionic resistance in kΩ.cm^2^
$\:{A}_{a}$: Membrane effective area
$\:{E}_{oc}$: Nernst reversible voltage, V
$\:T$: Operating temperature, Kelvin
$$\:{P}_{H}$$: Hydrogen pressure
$$\:{P}_{O}$$: Oxygen pressure
$$\:\:{P}_{W}$$: Water pressures
$\:R$: Universal gas constant (8.3145 J/mol/K)
$\:{D}_{f}$: Faraday constant (96 485 C/mol)
$\:{E}_{s}$: Reverse standard potential
$\:{N}_{c}$: Number of identical cells
$\:{V}_{out}$: Total system voltage, estimated
$\:{V}_{m}$: Real measured output voltage
$\:\varDelta\:{B}_{t}$: Gibbs free energy
$\:{H}_{M}$: Molar mass of hydrogen
$\:{O}_{\text{M}}$: Molar mass of oxygen
$$\:{C}_{a}$$: Anode constant
$\:{C}_{O}$: Oxygen molar constant
$\:{C}_{H}$: Hydrogen molar constant
$$\:{A}_{v}$$: Anode volume
$\:{F}_{{IN-H}_{2}}$: Hydrogen inlet flow rate
$\:{F}_{{OU-H}_{2}}$: Hydrogen outlet flow rate
$\:{F}_{{RH}_{2}}$: Real combined hydrogen
$\:{K}_{R}$: Combined hydrogen constant
$\:{C}_{{H}_{2}O}$: Water molar constant
$\:{F}_{f}$: Fuel flow rate
$\:{\:F}_{{H}_{2}}$: Hydrogen flow rate
$\:{F}_{{O}_{2}}$: Oxygen flow rate
$\:{R}_{{T}_{H-O}}$: Hydrogen to oxygen ratio
$\:\nabla\:$: Gradient descent factor of alpha, beta and gamma
$\:X\left(t\right)$: Current position of particles
$\:\rho\:$: Propagation of particles
$\:{\Delta\:}$: Difference between particles position and human position
$$\:\text{c}$$: Constant
$\:W$: Outdoor walking speed of adults
$\:{A}_{h}$: Human walking area
$\:rand$: Random value between 0 and 1
$\:{A}_{p}$: Area of propagation of particles
$\:{X}_{T}\left(t\right)$: Average of total position
$\:v$: Particles speed, equals 16000 for 𝛼 particles, 270000 for 𝛽 particles and 300000 for γ particles
$$\:{N}_{pop}$$: Population size
$$\:m$$: Problem dimension
$\:Max\_Iter$: Maximum number of iterations
$\:{U}_{{O}_{\text{p}\text{t}}}$: Fuel utilization factor
$\:{K}_{{H}_{2}}$: Gain constant values of $\:{H}_{2}$, kmol/(atm.s)
$\:{K}_{{H}_{2}O}$: Gain constant values of $\:{\:H}_{2}O$, kmol/(atm.s)
$\:{K}_{{O}_{2}}$: Gain constant values of $\:{O}_{2}$, kmol/(atm.s)
$\:{\tau\:}_{f}$: Time constant of fuel process, s
$\:{\tau\:}_{{H}_{2}}$: Time constant of $\:{H}_{2},\:\text{s}$
$\:{\tau\:}_{{H}_{2}O}$: Time constant of $\:{\:H}_{2}O$, s
$\:{\tau\:}_{{O}_{2}}$: Time constant of $\:{O}_{2}$, s
$$\:{\tau\:}_{r}$$: Electrical response, s
$\:{P}_{load}$: Active power of the load, W
$\:{P}_{Grid}$: Active power of the grid, W
$\:{L}_{SMES}$: Magnet coil inductance, H
$\:{V}_{SMES}$: Voltage of the SMES, V
$\:{I}_{SMES}$: Current of the SMES, A
$\:{P}_{SMES}$: Power of the SMES, W
$\:{E}_{SMES}$: Energy stored in the SMES
$\:{P}_{SOFC}$: Output power of SOFCs stack, W
$\:{V}_{c}$: Output voltage of SOFC cell, V
